# Association between frailty and kidney stone among U.S. adults using data from NHANES

**DOI:** 10.1097/MD.0000000000045290

**Published:** 2025-10-17

**Authors:** Jie Liu, Hege Bian, Yue Wang, Kun Liu

**Affiliations:** a Department of Intensive Care Unit (ICU), The Affiliated Qingyuan Hospital (Qingyuan People’s Hospital), Guangzhou Medical University, Qingyuan, Guangdong Province, China; b Supply Chain Department, Hefei BOE Hospital, Hefei, China; c Department of Science and Education, Hefei BOE Hospital, Hefei, China; d Department of Surgery, The Third Affiliated Hospital of Anhui Medical University (The First People’s Hospital of Hefei), Hefei, Anhui Province, China.

**Keywords:** epidemiology, frailty, kidney stones, NHANES, risk factors

## Abstract

This study aimed to investigate the association between frailty and kidney stone prevalence among U.S. adults. We conducted a cross-sectional analysis using data from National Health and Nutrition Examination Survey 2017 to 2020. Frailty was assessed using a 49-item frailty index (FI) based on the cumulative deficit model and categorized into robust (FI ≤ 0.10), pre-frail (0.10 < FI < 0.25), and frail (FI ≥ 0.25). Kidney stone history was self-reported. Weighted logistic regression models were used to estimate the association between frailty and kidney stones, with adjustments for demographic, socioeconomic, lifestyle, comorbidity, and biochemical covariates. A total of 26,454 participants were included, with 2530 (9.6%) reporting a history of kidney stones. The FI was positively associated with kidney stone risk (OR per 0.01 increase = 1.024; 95% CI: 1.016–1.032; *P* < .0001). Compared with robust individuals, the adjusted odds ratios for kidney stones were 1.509 (95% CI: 1.310–1.740) for the pre-frail group and 1.852 (95% CI: 1.472–2.331) for the frail group (*P* for trend <.0001). RCS analysis confirmed a nonlinear dose-response relationship. Subgroup analyses revealed stronger associations in individuals <60 years, females, and those with lower socioeconomic status, with a significant interaction by age (*P* for interaction <.0001). Frailty is independently associated with an increased risk of kidney stones in U.S. adults, particularly among younger and socioeconomically vulnerable populations. However, due to the cross-sectional nature of this study, causality cannot be inferred. These findings highlight the potential value of frailty screening in kidney stone prevention strategies and warrant further prospective and mechanistic research.

## 1. Introduction

Over recent decades, the global incidence of kidney stones has exhibited a marked and steady increase. In the United States, the prevalence has risen significantly from 3.2% in the 1980s to approximately 9.6% in recent years.^[1,2]^ Symptomatic urolithiasis is notably more common in men, with peak incidence typically observed between the ages of 40 and 60 years, whereas in women, the peak tends to occur around the age of 50.^[3,4]^ This upward trend has been largely attributed to the growing prevalence of obesity, diabetes mellitus (DM), metabolic syndrome, and Western dietary patterns.^[5–7]^

Frailty, a geriatric syndrome marked by reduced physiological reserve and vulnerability to stressors, has been linked to adverse outcomes in cardiovascular, respiratory, and renal diseases.^[8–14]^ However, its potential relationship with kidney stone disease has not been systematically examined, despite plausible shared mechanisms such as inflammation, metabolic disturbances, and reduced mobility. Several underlying mechanisms suggest a plausible connection. Frail individuals often experience sarcopenia, low-grade systemic inflammation, malnutrition, and reduced mobility^[15]^ – factors that may contribute to altered urinary biochemistry, dehydration, and metabolic disturbances conducive to stone formation.^[16]^ Moreover, frailty is frequently accompanied by comorbidities such as diabetes, obesity, and metabolic syndrome,^[17–19]^ which are well-established risk factors for kidney stones. A related study indicates that weakness may be a risk factor for urolithiasis in patients with diabetes.^[20]^ However, to date, no nationally representative studies have systematically examined this relationship in the general population.

Given the increasing burden of both frailty and kidney stone disease in aging populations, and the lack of population-based evidence regarding their potential interrelationship, further investigation is warranted. The National Health and Nutrition Examination Survey (NHANES) provides a unique opportunity to examine this association in a nationally representative sample of U.S. adults, with comprehensive data on demographic characteristics, health status, and lifestyle factors. Therefore, the aim of this study was to evaluate the association between frailty and the prevalence of kidney stones among American adults using NHANES 2017 to 2020 data. We hypothesized that higher frailty index (FI) scores would be independently associated with an increased likelihood of kidney stone history, even after adjusting for known risk factors such as age, sex, and, comorbidities.

## 2. Methods

### 2.1. Study population and design

This cross-sectional study utilized data from the 2017 to 2018 and 2019 to 2020 cycles of the NHANES, a nationwide program conducted by the Centers for Disease Control and Prevention (CDC) and the National Center for Health Statistics. NHANES is designed to assess the health and nutritional status of the non-institutionalized U.S. population through a complex, multistage probability sampling method, with publicly available data released in biennial cycles. All participants provided written informed consent, and the NHANES protocol was approved by the CDC’s institutional review board. In accordance with institutional policy, our local ethics committee waived review for this secondary analysis of de-identified public-use data.

**Figure 1.**
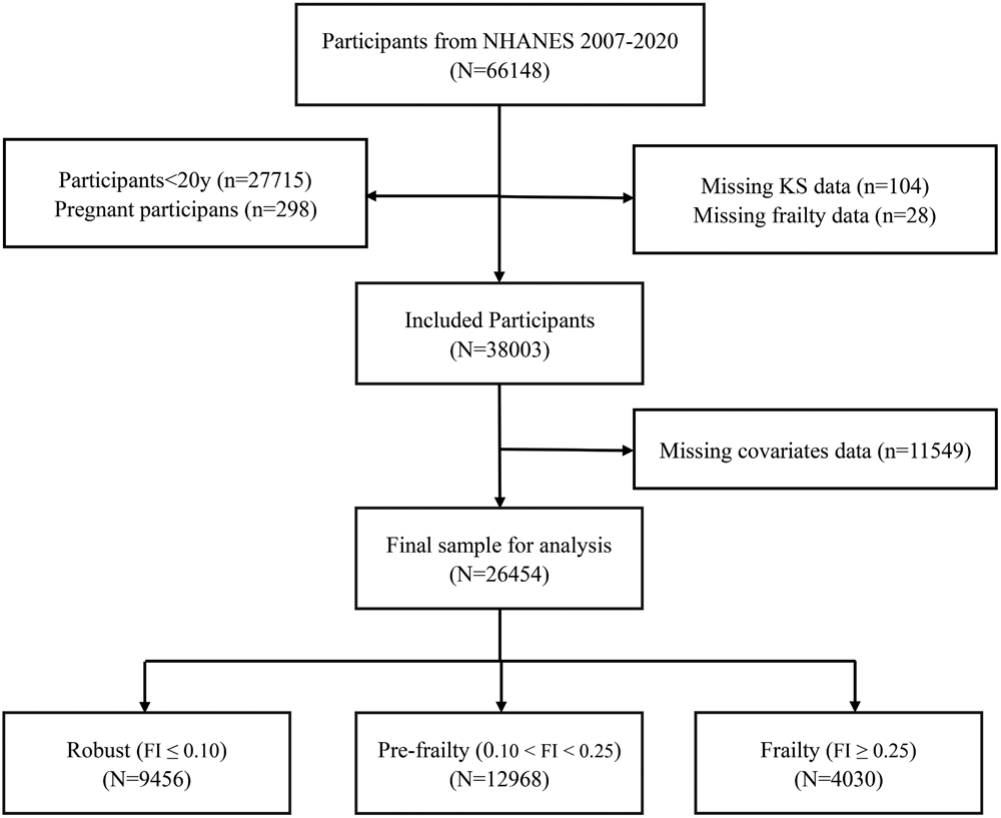
Flowchart of participant selection from NHANES 2017–2020. NHANES = National Health and Nutrition Examination Survey.

As illustrated in Figure 1, a total of 66,148 individuals participated across the 2 NHANES cycles. We excluded participants under the age of 20 years (n = 27,715) and pregnant individuals (n = 298). Additional exclusions included those lacking data on kidney stone history (n = 104), FI variables (n = 28), and key covariates (n = 11,549). After applying these criteria, a final analytic sample of 26,454 adult participants was included in the study.

### 2.2. Frailty index

The FI was operationalized using the cumulative deficit model, calculated as the ratio of the number of health deficits present in an individual to the total number of potential deficits included in the model. A higher FI score reflects greater frailty. The standardization of this methodology has been shown to be reproducible and is widely employed to capture health-related vulnerabilities in aging populations.^[21]^ To ensure the reliability of the FI, participants were required to have responses for at least 80% of the 49 selected frailty-related variables. Prior studies have demonstrated that using approximately 30 to 40 variables randomly selected from different health domains is sufficient for predicting adverse health outcomes.^[22,23]^ In this study, we included 49 variables spanning 7 key domains: cognitive function (1 item), functional dependence (16 items), depressive symptoms (7 items), comorbid conditions (13 items), healthcare utilization (5 items), anthropometric measures (1 item), and laboratory biomarkers (6 items).^[24]^ Each variable was scored on a scale from 0 to 1 based on the degree of deficit, with 0 indicating no deficit and 1 representing full expression of the deficit. For continuous variables, cutoffs were determined based on clinical guidelines or established thresholds in the literature.

The final FI score was obtained by summing the individual scores and dividing by the total number of variables assessed. Participants were then categorized into 3 frailty levels based on established cutoff values^[25–27]^: robust (FI ≤ 0.10), pre-frail (0.10 < FI < 0.25), and frail (FI ≥ 0.25).

### 2.3. Determination of kidney stones

Information regarding kidney stone history was obtained through a structured questionnaire administered as part of the NHANES kidney conditions section. Trained interviewers conducted face-to-face interviews using a standardized protocol to ensure data reliability. Participants were classified as having a history of kidney stones if they responded “yes” to the question, “Have you ever had kidney stones?” This self-reported measure has been commonly used in previous NHANES-based epidemiologic studies and is considered a valid approach for identifying symptomatic kidney stone cases in large-scale surveys.

### 2.4. Covariables

To account for potential confounding in the relationship between frailty and kidney stone prevalence, a comprehensive set of covariates was included in the multivariable-adjusted models. Demographic variables comprised age (as a continuous variable), sex (male or female), race/ethnicity, educational level, and poverty income ratio (PIR) as an indicator of socioeconomic status. Social and lifestyle characteristics included marital status (living alone vs cohabiting), smoking status (never, former, or current), and alcohol consumption (never/former, light, moderate, or heavy). To reflect hepatic and renal function, laboratory biomarkers were incorporated, including alanine aminotransferase (ALT), aspartate aminotransferase (AST), serum creatinine (Cre), blood urea nitrogen (BUN), uric acid (UA), serum phosphorus (P), and serum calcium (Ca). In addition, major comorbidities were considered: hypertension, DM, hyperlipidemia, and cardiovascular disease (CVD). Hypertension was defined as a prior diagnosis, current use of antihypertensive medications, or measured systolic/diastolic blood pressure ≥ 140/90 mm Hg during the NHANES Mobile Examination Center assessment. Diabetes status was categorized as diabetic, borderline, or diabetic. Participants were classified as diabetic if they had a previous diagnosis, were using antidiabetic medications or insulin, had a fasting glucose level > 126 mg/dL, or a 2-hour plasma glucose level ≥ 200 mg/dL during an Oral Glucose Tolerance Test. Those with impaired fasting glucose or impaired glucose tolerance were defined as borderline diabetic; others were classified as non-diabetic. Hyperlipidemia was defined by a history of diagnosis, use of lipid-lowering medications, or TC ≥ 240 mg/dL. CVD was defined as a history of myocardial infarction, angina, coronary heart disease, or congestive heart failure. Definitions and measurement procedures for all variables are publicly available on the NHANES website (www.cdc.gov/nchs/nhanes/).

### 2.5. Statistical analysis

All analyses were conducted using appropriate sampling weights, strata, and primary sampling units to account for the complex, multistage probability design of NHANES, ensuring national representativeness. Continuous variables were presented as weighted means with standard errors, while categorical variables were expressed as weighted counts or percentages. Group differences were assessed using survey-weighted *t*-tests for continuous variables and Rao–Scott χ^2^ tests for categorical variables.

To explore the association between frailty and kidney stone prevalence, survey-weighted logistic regression models were employed. Three hierarchical models were constructed. Model 1 was unadjusted. Model 2 included demographic and socioeconomic covariates: age, sex, race/ethnicity, marital status, educational attainment, PIR, and BMI. Model 3 further adjusted for lifestyle factors (alcohol use, smoking status), clinical comorbidities (DM, hypertension, CVD, hyperlipidemia), and laboratory biomarkers relevant to renal and metabolic function (UA, P, Ca, Cre, ALT, AST, and BUN).

To assess the potential non-linear dose-response relationship between frailty and kidney stones, restricted cubic spline (RCS) models were applied using the FI as a continuous variable. Subgroup analyses were performed to evaluate the consistency of associations across different demographic and clinical strata. All statistical analyses were conducted using R software (version 4.2.0), and a two-sided *P*-value < .05 was considered indicative of statistical significance.

## 3. Results

### 3.1. Baseline characteristics

A total of 26,454 participants were included in the final analysis, among whom 2530 (9.56%) reported a history of kidney stones (Table 1). Compared with those without kidney stones, participants with a history of stones were older (52.91 vs 46.83 years, *P* < .0001) and had higher BMI (30.71 vs 28.95 kg/m^2^, *P* < .0001). They also exhibited higher serum levels of Cre, UA, and BUN, along with lower serum phosphorus (all *P* < .0001). The kidney stone group had a higher proportion of males (55.60% vs 48.70%), non-Hispanic Whites (78.23% vs 68.21%), individuals with obesity (BMI ≥ 30 kg/m^2^: 48.06% vs 36.64%), and comorbidities including hypertension (53.32% vs 36.03%), DM (24.78% vs 13.16%), hyperlipidemia (78.88% vs 69.63%), and CVD (15.32% vs 7.89%) (all *P* < .0001). In terms of frailty, individuals with kidney stones had significantly higher FI scores (0.173 vs 0.135, *P* < .0001), and were more likely to be classified as frail (FI ≥ 0.25: 19.53% vs 10.49%) or pre-frail (53.63% vs 46.84%).

**Table 1:** Baseline characteristics of participants according to kidney stone status in NHANES 2017–2020.

Variables	Overall	Non-kidney stones	Kidney stones	*P* value
No of participants	26,454	23,924	2530	
Age, yr	47.43 ± 0.25	46.83 ± 0.26	52.91 ± 0.36	<.0001
PIR	3.05 ± 0.04	3.05 ± 0.04	3.04 ± 0.06	.93
BMI, kg/m^2^	29.13 ± 0.08	28.95 ± 0.08	30.71 ± 0.15	<.0001
ALT, U/L	25.30 ± 0.15	25.22 ± 0.16	25.95 ± 0.39	.08
AST, U/L	25.26 ± 0.12	25.22 ± 0.13	25.63 ± 0.29	.21
Cre, mg/dL	0.89 ± 0.00	0.88 ± 0.00	0.93 ± 0.01	<.0001
UA, mg/dL	5.44 ± 0.01	5.42 ± 0.01	5.65 ± 0.04	<.0001
BUN, mg/dL	13.69 ± 0.07	13.57 ± 0.07	14.75 ± 0.16	<.0001
P, mg/dL	3.73 ± 0.01	3.73 ± 0.01	3.65 ± 0.02	<.0001
Ca, mg/dL	9.40 ± 0.01	9.40 ± 0.01	9.38 ± 0.01	.07
Frailty score	0.139 ± 0.001	0.135 ± 0.001	0.173 ± 0.003	<.0001
Age group, %				
<60	74.00	75.13	63.78	<.0001
≥60	26.00	24.87	36.22	
Sex, %				
Female	50.62	51.30	44.40	<.0001
Male	49.38	48.70	55.60	
Race/ethnicity, %				
Mexican American	8.04	8.27	5.94	<.0001
Non-Hispanic White	69.20	68.21	78.23	
Non-Hispanic Black	10.11	10.63	5.37	
Other races	12.64	12.88	10.46	
Marital status, %				
Solitude	36.40	37.07	30.26	<.0001
Cohabitation	63.60	62.93	69.74	
PIR group, %				
<1.3	20.77	20.92	19.38	.08
1.3–3.5	35.03	34.76	37.51	
≥3.5	44.20	44.32	43.12	
Educational level, %				
Below high school	14.56	14.48	15.21	.52
High school	22.54	22.49	23.05	
Above high school	62.90	63.03	61.74	
BMI group, %				
<25 kg/m^2^	29.18	30.22	19.75	<.0001
25–30 kg/m^2^	33.05	33.15	32.19	
≥30 kg/m^2^	37.77	36.64	48.06	
Smoke status, %				
Never	55.41	56.09	49.27	<.0001
Former	25.14	24.55	30.45	
Now	19.45	19.36	20.28	
Alcohol user, %				
Never	10.35	10.39	9.94	<.0001
Former	12.66	12.11	17.60	
Mild	37.20	36.89	40.03	
Moderate	17.98	18.17	16.30	
Heavy	21.82	22.44	16.12	
Hypertension, %				
No	62.26	63.97	46.68	<.0001
Yes	37.74	36.03	53.32	
DM, %				
No	77.23	78.38	66.74	<.0001
Borderline	8.46	8.46	8.48	
Yes	14.31	13.16	24.78	
Hyperlipidemia				
No	29.46	30.37	21.12	<.0001
Yes	70.54	69.63	78.88	
CVD, %				
No	91.37	92.11	84.68	<.0001
Yes	8.63	7.89	15.32	
Frailty status				
Robust (FI ≤ 0.10)	41.10	42.67	26.84	<.0001
Pre-frail (0.10 < FI < 0.25)	47.51	46.84	53.63	
Frail (FI ≥ 0.25)	11.39	10.49	19.53	

Continuous variables are expressed as mean ± standard error, while categorical variables are presented as weighted percentages. To compare groups, weighted linear regression analysis was used for continuous variables, and weighted chi-square tests were applied for categorical variables. Statistical significance was defined as a *P*-value <.05.

ALT = alanine aminotransferase, AST = aspartate aminotransferase, BMI = body mass index, BUN = blood urea nitrogen, Ca = serum calcium, Cre = creatinine, CVD = cardiovascular diseases, DM = diabetes mellitus, NHANES = National Health and Nutrition Examination Survey, P = serum phosphate, PIR = poverty income ratio, UA = uric acid.

In addition, baseline characteristics stratified by frailty categories (robust, pre-frail, and frail) are presented in Table S1 (Supplemental Digital Content, https://links.lww.com/MD/Q355). Significant differences were observed across frailty groups for most demographic, clinical, and biochemical variables.

### 3.2. Association between frailty and kidney stones

Table 2 showed a consistent positive association between frailty and kidney stone prevalence. When modeled as a continuous variable (FI × 100), each 0.01 increase in frailty was associated with a 2.4% higher odds of kidney stones in the fully adjusted model (OR = 1.024; 95% CI: 1.016–1.032; *P* < .0001), with similar associations observed in the unadjusted and partially adjusted models.

**Table 2:** Association between frailty and kidney stone prevalence using logistic regression models.

Exposure	Model 1		Model 2		Model 3	
	OR (95% CI)	*P* value	OR (95% CI)	*P* value	OR (95% CI)	*P* value
Frailty index × 100	1.039 (1.034,1.044)	<.0001	1.031 (1.024,1.037)	<.0001	1.024 (1.016,1.032)	<.0001
Frailty status						
Robust (FI ≤ 0.10)	Ref		Ref		Ref	
Pre-frail (0.10 < FI < 0.25)	1.821 (1.601,2.071)	<.0001	1.651 (1.449,1.881)	<.0001	1.509 (1.310,1.740)	<.0001
Frail (FI ≥ 0.25)	2.961 (2.496,3.513)	<.0001	2.326 (1.908,2.835)	<.0001	1.852 (1.472,2.331)	<.0001
*P* for trend	<.0001		<.0001		<.0001	

Model 1: no covariates; Model 2: with age, sex, race, marital status, educational level, PIR, and BMI status; Model 3: with age, sex, race, marital status, educational level, PIR, BMI status, alcohol user, smoke status, DM, hypertension, CVD, hyperlipidemia, UA, P, Ca, Cre, ALT, AST, and BUN.

ALT = alanine aminotransferase, AST = aspartate aminotransferase, BMI = body mass index, BUN = blood urea nitrogen, Ca = serum calcium, Cre = creatinine, CVD = cardiovascular diseases, DM = diabetes mellitus, P = serum phosphate, PIR = poverty income ratio, UA = uric acid.

When frailty was analyzed categorically, individuals classified as pre-frail and frail showed significantly increased odds of kidney stones compared to robust participants. In Model 3, the adjusted ORs were 1.509 (95% CI: 1.310–1.740) for the pre-frail group and 1.852 (95% CI: 1.472–2.331) for the frail group (both *P* < .0001). A clear dose-response trend was observed across frailty categories (*P* for trend <.0001).

**Figure 2.**
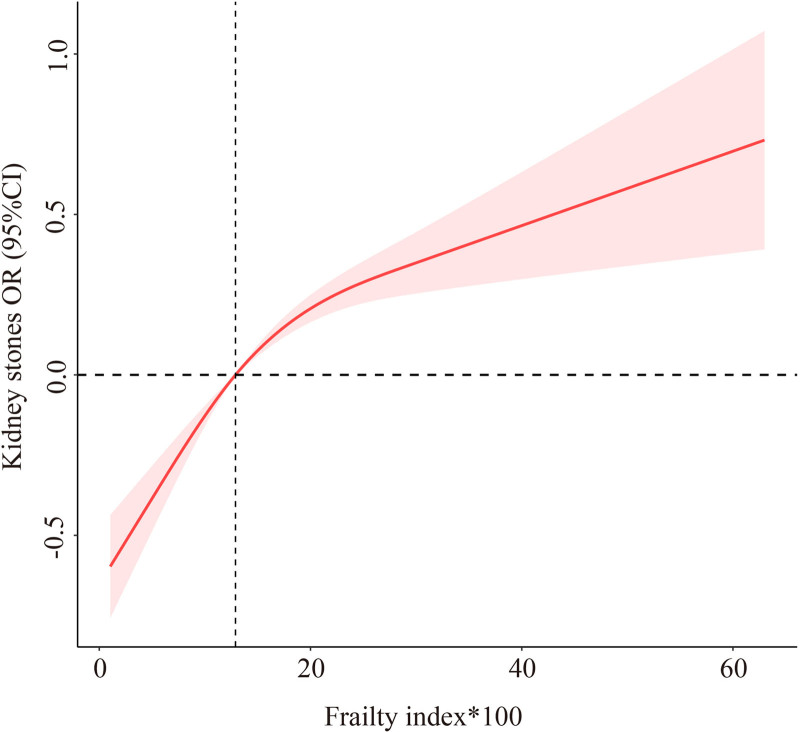
Restricted cubic spline showing the dose-response relationship between frailty index and kidney stone prevalence.

RCS analysis demonstrated a nonlinear positive association between the FI and the odds of kidney stones (Fig. 2). As frailty increased, the risk of kidney stones rose progressively, with a steeper slope observed at higher frailty levels. The 95% CI indicated statistical significance throughout most of the range, supporting a graded dose-response relationship between frailty burden and kidney stone prevalence.

### 3.3. Subgroup analysis

**Figure 3.**
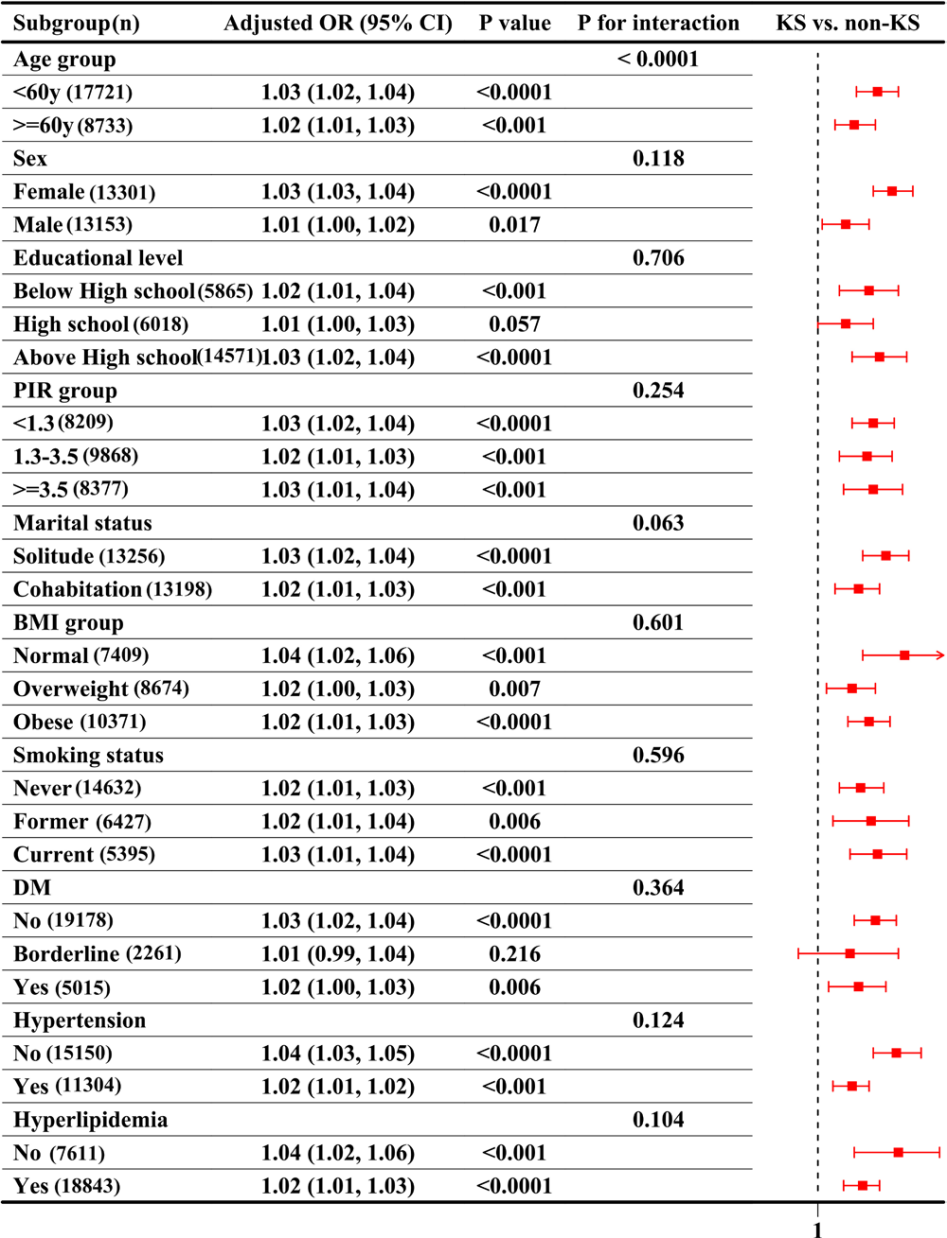
Subgroup analysis of the association between frailty index (continuous) and kidney stone prevalence (forest plot).

Subgroup analyses demonstrated that the positive association between frailty and kidney stone prevalence remained robust across nearly all strata (Fig. 3, Table 3). When modeled as a continuous variable, the FI was significantly associated with higher odds of kidney stones in all subgroups (all *P* < .05), with no substantial effect modification by sex, education, BMI, smoking, diabetes, hypertension, or hyperlipidemia (*P* for interaction >.05 for all). However, interaction by age was statistically significant (*P* for interaction <.0001), with a slightly stronger association observed in participants under 60 years.

**Table 3:** Subgroup analysis of the association between frailty status and kidney stones across demographic and clinical strata.

Subgroup	Participants (n)	Robust (FI ≤ 0.10)	Pre-frail (0.10 < FI < 0.25)	Frail (FI ≥ 0.25)	*P* for trend	*P* for interaction
Age group						
<60 yr	17,721	Ref	1.506 (1.270, 1.787)	2.050 (1.548, 2.715)	<.0001	<.0001
≥60 yr	8733	Ref	1.411 (1.123, 1.773)	1.542 (1.136, 2.093)	.005	
Sex						
Female	13,301	Ref	1.987 (1.578, 2.502)	2.752 (2.060, 3.675)	<.0001	.031
Male	13,153	Ref	1.244 (1.020, 1.517)	1.316 (0.965, 1.794)	.031	
Educational level						
Below high school	5865	Ref	1.598 (1.089, 2.343)	2.059 (1.320, 3.211)	.001	.276
High school	6018	Ref	1.780 (1.267, 2.502)	1.730 (1.093, 2.738)	.006	
Above high school	14,571	Ref	1.401 (1.130, 1.736)	1.881 (1.328, 2.665)	<.001	
PIR group						
<1.3	8209	Ref	2.048 (1.512, 2.774)	2.551 (1.841, 3.536)	<.0001	.092
1.3–3.5	9868	Ref	1.295 (1.041, 1.611)	1.693 (1.222, 2.347)	.002	
≥3.5	8377	Ref	1.530 (1.191, 1.966)	1.737 (1.153, 2.616)	<.001	
Marital status						
Solitude	13,256	Ref	1.725 (1.351, 2.202)	2.441 (1.747, 3.411)	<.0001	.109
Cohabitation	13,198	Ref	1.442 (1.206, 1.725)	1.598 (1.188, 2.148)	<.001	
BMI group						
Normal	7409	Ref	1.651 (1.219, 2.236)	2.665 (1.436, 4.946)	<.001	.40
Overweight	8674	Ref	1.360 (1.077, 1.716)	1.572 (1.049, 2.354)	.009	
Obese	10,371	Ref	1.572 (1.263, 1.955)	1.881 (1.391, 2.542)	<.0001	
Smoking status						
Never	14,632	Ref	1.526 (1.221, 1.907)	1.740 (1.292, 2.343)	<.0001	.207
Former	6427	Ref	1.628 (1.222, 2.169)	1.755 (1.063, 2.897)	.012	
Current	5395	Ref	1.272 (0.952, 1.699)	2.070 (1.346, 3.182)	.002	
DM						
No	19,178	Ref	1.553 (1.317, 1.832)	1.985 (1.477, 2.667)	<.0001	.732
Borderline	2261	Ref	1.036 (0.647, 1.659)	0.950 (0.488, 1.848)	.938	
Yes	5015	Ref	1.547 (0.966, 2.475)	1.931 (1.135, 3.283)	.012	
Hypertension						
No	15,150	Ref	1.633 (1.372, 1.944)	2.442 (1.769, 3.369)	<.0001	.701
Yes	11,304	Ref	1.165 (0.940, 1.444)	1.374 (1.038, 1.818)	.027	
Hyperlipidemia						
No	7611	Ref	1.686 (1.262, 2.251)	2.240 (1.221, 4.110)	<.001	.08
Yes	18,843	Ref	1.434 (1.218, 1.690)	1.752 (1.384, 2.218)	<.0001	

Model 1: no covariates; Model 2: with age, sex, race, marital status, educational level, PIR, and BMI status; Model 3: with age, sex, race, marital status, educational level, PIR, BMI status, alcohol user, smoke status, DM, hypertension, CVD, hyperlipidemia, UA, P, Ca, Cre, ALT, AST, and BUN.

ALT = alanine aminotransferase, AST = aspartate aminotransferase, BMI = body mass index, BUN = blood urea nitrogen, Ca = serum calcium, Cre = creatinine, CVD = cardiovascular diseases, DM = diabetes mellitus, P = serum phosphate, PIR = poverty income ratio, UA = uric acid.

When frailty was analyzed categorically, both pre-frail and frail individuals exhibited consistently elevated risks across subgroups. For example, among females, the ORs for kidney stones were 1.99 (95% CI: 1.58–2.50) for pre-frail and 2.75 (95% CI: 2.06–3.68) for frail individuals; in males, the corresponding ORs were 1.24 and 1.32, respectively. Similar patterns were observed across BMI and income strata. In the low-income group (PIR < 1.3), frail individuals had over 2.5 times the odds of kidney stones compared to robust participants (OR = 2.55; 95% CI: 1.84–3.54).

## 4. Discussion

In this nationally representative cross-sectional study, we found that frailty was independently associated with a higher prevalence of kidney stones, even after adjustment for demographic, clinical, and biochemical factors. These results align with and extend previous findings in specific patient populations, such as individuals with diabetes,^[20]^ by demonstrating this association in the general adult population. Our study is among the first to use NHANES data to evaluate this relationship, providing nationally representative evidence that frailty may be an underrecognized risk factor for nephrolithiasis. However, that study focused exclusively on DM populations. Our research broadens this scope by demonstrating a significant association between frailty and kidney stone prevalence in the general adult population, independent of DM status. Furthermore, while prior studies have primarily concentrated on the impact of frailty on surgical outcomes in urolithiasis patients,^[28]^ our study is among the first to utilize NHANES data to explore the epidemiological relationship between frailty and kidney stones.

Although the underlying mechanisms connecting frailty and an elevated risk of kidney stones have not been fully elucidated, several plausible pathways may explain this association. One important factor is reduced physical activity. By definition, frail individuals exhibit markedly diminished mobility and muscle strength, often leading to sedentary behavior or prolonged periods of immobility. Such inactivity has been implicated in increased bone resorption and consequent absorptive hypercalciuria^[29,30]^ – a well-recognized risk factor for calcium-based kidney stones. Another contributing factor may be dietary modifications commonly recommended for frail individuals. To prevent protein-energy malnutrition and maintain nitrogen balance, increased dietary protein intake is often encouraged.^[31]^ However, high-protein diets have been shown to elevate urinary calcium excretion and the risk of stone formation.^[32]^ This is likely due to acid generation during protein catabolism, which induces buffering through bone mineral release, enhances intestinal calcium absorption, and increases glomerular filtration – all of which contribute to calcinuria.^[33]^ Collectively, these physiological changes – reduced activity, bone demineralization, and altered nutrient metabolism – may partly account for the observed association between frailty and kidney stone formation.

A significant interaction by age was observed in our subgroup analysis, with a stronger association between frailty and kidney stones noted among participants under 60 years of age. This finding is noteworthy, as frailty is traditionally considered a geriatric syndrome, yet our results suggest that early-onset or “premature frailty” may exert substantial health impacts even in younger adults. Several possible explanations may account for this age-related difference. Younger individuals with frailty may experience more active metabolic disturbances, such as insulin resistance, sarcopenic obesity, or inflammation-driven bone resorption, all of which are established contributors to nephrolithiasis pathogenesis.^[34]^ In contrast, older adults may have a higher baseline prevalence of comorbid conditions that independently influence kidney stone risk, potentially attenuating the relative contribution of frailty itself. Moreover, physiological compensation mechanisms may differ with age, as younger individuals may be more sensitive to the metabolic burden imposed by frailty. Our findings align with recent research highlighting the clinical relevance of frailty in middle-aged populations. For example, a study based on the UK Biobank found that frailty in adults aged 40 to 59 was significantly associated with incident multimorbidity and increased healthcare utilization.^[35]^ These results collectively underscore the need to recognize and address frailty as a meaningful risk factor in younger adults, particularly given its modifiable nature. Moreover, the stronger association observed in women may partly reflect sex-related differences in stone pathophysiology. Estrogen has been suggested to modulate calcium and oxalate metabolism, with declining estrogen levels after menopause contributing to increased stone risk.^[36]^ Moreover, women may have distinct urinary biochemical profiles, including lower citrate excretion and higher urinary tract infection prevalence, both of which predispose to nephrolithiasis.^[37]^ These mechanisms may amplify the impact of frailty-related metabolic and inflammatory disturbances on stone formation in women.

Several limitations should be acknowledged when interpreting our findings. First, due to the cross-sectional design of NHANES, causal relationships cannot be established. Specifically, we cannot determine whether frailty predisposes individuals to kidney stones, or whether recurrent kidney stones and their associated morbidity contribute to the development of frailty. Longitudinal studies are needed to confirm the temporal direction of this association. Second, kidney stone history was based on a single self-reported question, which is subject to recall bias and cannot capture asymptomatic or undiagnosed stones. This underreporting could bias the results toward the null, suggesting that the true association between frailty and kidney stones may be underestimated in our study. Third, although we adjusted for a wide range of potential confounders, residual confounding from unmeasured variables – such as fluid intake patterns, stone composition, medication use (e.g., diuretics), or family history of urolithiasis – cannot be completely excluded. Fourth, the FI was constructed using available NHANES variables and may not fully capture all dimensions of frailty as defined in clinical settings. Moreover, although excluding participants with major comorbidities could be another approach to assess robustness, such exclusions would markedly reduce sample size and precision. Instead, we adjusted for these comorbidities in multivariable models to minimize confounding. Lastly, while NHANES provides nationally representative estimates, the findings may not be generalizable to institutionalized populations or non-U.S. settings.

## 5. Conclusions

Frailty is independently associated with kidney stone history in U.S. adults. Given the cross-sectional design, causality cannot be inferred, and prospective studies are needed to clarify temporal relationships and mechanisms.

## Acknowledgments

Thanks to all NHANES participants and staff.

## Author contributions

**Conceptualization:** Jie Liu, Hege Bian, Yue Wang, Kun Liu.

**Data curation:** Jie Liu, Hege Bian, Yue Wang, Kun Liu.

**Formal analysis:** Jie Liu, Hege Bian, Kun Liu.

**Investigation:** Jie Liu, Kun Liu.

**Methodology:** Jie Liu, Yue Wang, Kun Liu.

**Project administration:** Jie Liu, Hege Bian, Yue Wang, Kun Liu.

**Resources:** Jie Liu, Hege Bian.

**Software:** Jie Liu, Hege Bian, Yue Wang.

**Supervision:** Hege Bian, Yue Wang, Kun Liu.

**Validation:** Hege Bian, Yue Wang, Kun Liu.

**Visualization:** Kun Liu.

**Writing – original draft:** Jie Liu, Kun Liu.

**Writing – review & editing:** Jie Liu, Hege Bian, Yue Wang, Kun Liu.

## Supplementary Material


