# Supplementary material for: Association between frailty and kidney stone among U.S. adults using data from NHANES
Source: Medicine (Baltimore). 2025 Oct 17;104(42):e45290. doi: 10.1097/MD.0000000000045290 (PMC12537269; doi:10.1097/MD.0000000000045290)
Supplement: Supplementary file 1 [file medi-104-e45290-s001.docx]

**Table S1.** Baseline characteristics of participants according to frailty status in NHANES 2017–2020.

| **Variables** | **Overall** | **Frailty** | **None- Frailty** | **P value** |
| --- | --- | --- | --- | --- |
| Age, years | 47.43±0.25 | 58.52±0.34 | 46.00±0.26 | < 0.0001 |
| PIR | 3.05±0.04 | 2.27±0.05 | 3.15±0.04 | < 0.0001 |
| BMI, kg/m^2^ | 29.13±0.08 | 32.31±0.18 | 28.72±0.08 | < 0.0001 |
| ALT, U/L | 25.30±0.15 | 24.90±0.50 | 25.35±0.15 | 0.37 |
| AST, U/L | 25.26±0.12 | 26.43±0.43 | 25.11±0.11 | 0.003 |
| Cre, mg/dL | 0.89±0.00 | 1.02±0.02 | 0.87±0.00 | < 0.0001 |
| UA, mg/dL | 5.44±0.01 | 5.66±0.03 | 5.42±0.01 | < 0.0001 |
| BUN, mg/dL | 13.69±0.07 | 16.19±0.17 | 13.37±0.07 | < 0.0001 |
| P, mg/dL | 3.73±0.01 | 3.78±0.01 | 3.72±0.01 | < 0.0001 |
| Ca, mg/dL | 9.40±0.01 | 9.36±0.01 | 9.40±0.01 | < 0.001 |
| Frailty score | 0.14±0.00 | 0.33±0.00 | 0.11±0.00 | < 0.0001 |
| Age group, % |  |  |  | < 0.0001 |
| <60 | 74.00 | 52.61 | 76.75 |  |
| ≥60 | 26.00 | 47.39 | 23.25 |  |
| Sex, % |  |  |  | < 0.0001 |
| Female | 50.62 | 62.78 | 49.06 |  |
| Male | 49.38 | 37.22 | 50.94 |  |
| Race/ethnicity, % |  |  |  | < 0.0001 |
| Mexican American | 8.04 | 6.09 | 8.29 |  |
| Non-Hispanic White | 69.20 | 67.09 | 69.48 |  |
| Non-Hispanic Black | 10.11 | 14.89 | 9.50 |  |
| Other races | 12.64 | 11.94 | 12.73 |  |
| Marital status, % |  |  |  | < 0.0001 |
| Solitude | 2.56 | 36.17 | 18.66 |  |
| Cohabitation | 9.53 | 63.83 | 81.34 |  |
| PIR group, % |  |  |  | < 0.0001 |
| <1.3 | 20.77 | 35.57 | 18.87 |  |
| 1.3-3.5 | 35.03 | 40.25 | 34.36 |  |
| >=3.5 | 44.20 | 24.18 | 46.77 |  |
| Educational level, % |  |  |  | < 0.0001 |
| Below High school | 14.56 | 25.43 | 13.16 |  |
| High school | 22.54 | 27.02 | 21.97 |  |
| Above High school | 62.90 | 47.55 | 64.87 |  |
| BMI group, % |  |  |  | < 0.0001 |
| <25 kg/m^2^ | 29.18 | 16.82 | 30.77 |  |
| 25-30 kg/m^2^ | 33.05 | 28.84 | 33.59 |  |
| >=30 kg/m^2^ | 37.77 | 54.34 | 35.64 |  |
| Smoke status, % |  |  |  | < 0.0001 |
| Never | 55.41 | 39.46 | 57.46 |  |
| Former | 25.14 | 32.15 | 24.23 |  |
| Now | 19.45 | 28.39 | 18.30 |  |
| Alcohol user, % |  |  |  | < 0.0001 |
| Never | 10.35 | 12.37 | 10.09 |  |
| Former | 12.66 | 25.15 | 11.05 |  |
| Mild | 37.20 | 32.65 | 37.78 |  |
| Moderate | 17.98 | 13.22 | 18.59 |  |
| Heavy | 21.82 | 16.60 | 22.49 |  |
| Hypertension, % |  |  |  | < 0.0001 |
| No | 62.26 | 24.57 | 67.10 |  |
| Yes | 37.74 | 75.43 | 32.90 |  |
| DM, % |  |  |  | < 0.0001 |
| No | 77.23 | 48.78 | 80.88 |  |
| Borderline | 8.46 | 8.84 | 8.42 |  |
| Yes | 14.31 | 42.38 | 10.70 |  |
| Hyperlipidemia |  |  |  | < 0.0001 |
| No | 29.46 | 14.44 | 31.39 |  |
| Yes | 70.54 | 85.56 | 68.61 |  |
| CVD, % |  |  |  | < 0.0001 |
| No | 91.37 | 62.27 | 95.11 |  |
| Yes | 8.63 | 37.73 | 4.89 |  |
| Kidney stone status |  |  |  | < 0.0001 |
| No | 90.10 | 83.02 | 91.01 |  |
| Yes | 9.90 | 16.98 | 8.99 |  |

**Abbreviations:**

PIR, Poverty income ratio; BMI, Body mass index; ALT, alanine aminotransferase; AST, aspartate aminotransferase; Cre, creatinine; BUN, blood urea nitrogen; UA, uric acid; P, serum phosphate; Ca, serum calcium; DM, diabetes mellitus; CVD, Cardiovascular diseases.

**Statistical Analysis:**

Continuous variables are expressed as mean ± standard error, while categorical variables are presented as weighted percentages. To compare groups, weighted linear regression analysis was used for continuous variables, and weighted chi-square tests were applied for categorical variables. Statistical significance was defined as a p-value less than 0.05.
